# Optimizing nitrogen removal in advanced wastewater treatment using biological aerated filters

**DOI:** 10.3389/fbioe.2024.1463544

**Published:** 2024-11-28

**Authors:** Juan Li, Ziru Niu, Lei Li, Shuting Zhou

**Affiliations:** ^1^ Shaanxi Provincial Land Engineering Construction Group, Key Laboratory of Degraded and Unused Land Consolidation Engineering, Ministry of Natural Resources, Xi’an, China; ^2^ Shaanxi Engineering Research Center of Land Consolidation, Shaanxi Provincial Land Consolidation Engineering Technology Research Center, Xi’an, China; ^3^ Kweichow Moutai Winery (Group) Health Wine Co., LTD., Renhuai, China; ^4^ School of Environmental Science and Engineering, Suzhou University of Science and Technology, Suzhou, China

**Keywords:** immobilized biological aerated filter, total nitrogen removal, denitrification load, microbial community, advanced wastewater treatment

## Abstract

Reducing total nitrogen (TN) presents a significant challenge for numerous wastewater treatment facilities. In order to address this issue, the current study employed a biological aerated filter for the treatment of wastewater containing low nitrogen concentrations. Both lab-scale and pilot-scale biofilters were constructed to investigate the denitrification performance and maximum denitrification load. The findings indicated that the anaerobic denitrification process of established biofilm adhered to pseudo-first-order kinetics. The results of batch testing and continuous-flow experiments confirmed that the minimum hydraulic retention time (HRT) required for mature biofilm was determined to be 0.5 h. The optimal operating parameters were found to be as follows: influent NO_3_^−^-N concentration of 25 mg/L, HRT of 0.5 h, resulting in effluent TN levels below 1 mg/L. Under these conditions, the denitrifying load for the lab-scale I-BAF system was calculated to be 1.26 kg (TN)/(m^3^·d). Furthermore, it was observed that the maximum denitrifying load could reach 2.2 kg (TN)/(m^3^·d) when the influent NO_3_^−^-N concentration was increased to 50 mg/L while maintaining an HRT of 0.5 h. For the mature biofilter, the appropriate HRT ranged from 2 h to 0.5 h. Microbial diversity analysis revealed that the genus *Enterobacter* was dominant in all denitrification systems, followed by *Comamonas* and *Rhodococcus*. The operational parameters described in the paper could be recommended for a full-scale wastewater treatment facility.

## 1 Introduction

Currently, achieving low-pollutant effluent is a hot topic of study due to increasingly stringent discharge standards and water scarcity worldwide (Yaqoob et al., 2023). The primary challenge in this regard is to further reduce the concentration of total nitrogen (TN) (Omar et al., 2024). In China, the TN discharge concentration is limited to 15 mg/L (GB18918-2002) for wastewater treatment plants (WWTPs), while the European Union sets it at 10 mg/L. Furthermore, the World Health Organization (WHO) requires that nitrate concentration in drinking water should not exceed 10 mg/L (Su et al., 2020). TN in WWTP effluent is predominantly composed of nitrate, with the remaining nitrate originating from incomplete denitrification in mainstream wastewater treatment processes such as A^2^/O or oxidation ditches (Sheik et al., 2023). Currently, the TN concentration in secondary effluent from WWTPs in China ranges between 10 mg/L and 15 mg/L (Deng et al., 2021; Cui et al., 2023), and sometimes even reaches up to 25 mg/L (Liu et al., 2021). Such high nitrogen emissions can still cause eutrophication in many watersheds and pose a hazard to aquatic species. To address these issues, the Limited Denitrification Technology (LDT) has been proposed to further reduce the current TN concentration (Su et al., 2018).

The LDT method is primarily aimed at further reducing TN from low concentrations (15–25 mg/L) to ultra-low concentrations (<1 mg/L) in a stable manner. Applicable processes include short-term nitrification and denitrification, simultaneous nitrification-denitrification, aerobic denitrification, and anammox etc., (James and Vijayanandan, 2023). To achieve stable TN effluent standard, it may also be necessary to combine physical-chemical methods, such as sulfur autotrophic denitrification (Wang et al., 2023) and zero-valent iron denitrification processes (Zhou et al., 2024). These approaches can ensure ultra-low total nitrogen levels in the effluent while also promoting energy efficiency and reducing emissions. However, the key factor lies in whether the biological treatment stage can maintain a high TN removal efficiency. For example, if the biological treatment can reduce TN from 25 mg/L to 3 mg/L, then the sulfur autotrophic denitrification process can further reduce TN to around 1 mg/L.

Many studies assert that further reducing TN below 10 mg/L is an expensive process using mainstream technology (Chang and Wang, 2015), and some even suggest that achieving stable water quality with TN < 3 mg/L is impossible (Zhu et al., 2023). A large number of engineering applications indicate that traditional activated sludge processes can only ensure that the effluent TN remains stable at below 15 mg/L (Zhang et al., 2024). The biological filter process can achieve lower TN effluent concentration. However, commonly used media, such as volcanic rock and clay pellets, offer low biomass support, weak electron transfer capabilities, low microbial affinity, and are prone to clogging in practice (Yang et al., 2022). Additionally, the low effectiveness of inoculant strains also limits the ability of biological filters to further reduce TN effluent concentration (Simon et al., 2023). Therefore, the immobilized biological aerated filter (I-BAF) technology has been proposed to address these issues. In I-BAF, denitrifying microbes are immobilized on polyurethane foam (PUF) carriers, allowing the biofilm to develop a high-density biomass on the carriers and possess strong resistance to external stress (Gan et al., 2019). I-BAF can achieve ultra-low effluent nitrate concentrations under high loads and significantly reduce facility volume, demonstrating advantages in terms of economic efficiency and environmental friendliness. Several studies have aimed to elucidate the mechanisms and principles of I-BAF (Gan et al., 2020; Ye et al., 2005). However, there are few studies focused on ultra-low total nitrogen emissions and the maximum denitrifying load of biofilters, as observed in previous literature reviews.

In this study, both lab-scale and pilot-scale I-BAF systems were constructed to investigate the discharge of the lowest TN concentration using a mixed microbial consortium. Additionally, the microbial diversity characteristics of the biofilm under different operating conditions were investigated to elucidate the denitrifying performance of I-BAF. The operational parameters determined in this study could be applied to full-scale wastewater treatment facilities.

## 2 Materials and methods

### 2.1 Inoculation strain and assay methods

The inoculum strain used was a denitrifying microbial consortium (DMC), originally obtained from BIONETIX Co. (Canada) (Zhang et al., 2019). Synthetic wastewater was prepared based on the composition outlined in Table 1 (Gan et al., 2020). Prior to analysis, all samples were filtered through a 0.45 μm membrane. The TN concentration was defined as the sum of ammonia, nitrate, and nitrite (Cao et al., 2014).

**TABLE 1:** Experimental parameters for wastewater composition.

No.	Composition	Parameters	BAF_L_ (mg/L)			
			Adaption period*	Continous-flow test**	SDP test	LDL test
1	Nitrogen	KNO_3_	125 (initial TN)	25 (influent TN)	25 (influent TN)	25/50/100
2	Carbon source	Glucose	938 (initial COD)	125 (influent COD)	125 (influent COD)	125/250/500
3	Phosphorus	KH_2_PO_4_	6.25 (initial TP)	1.25 (influent TP)	1.25 (influent TP)	1.25/2.5/5
4	Other conditions	—	pH = 7.5, T = 25°C	pH = 7.5, T = 25°C	pH = 7.5, T = 25°C	pH = 7.5, T = 25°C
5	Trace elements	MgCl_2_·6H_2_O (20.3 mg/L), CaCl_2_ (1 mg/L), NiCl_2_·6H_2_O (0.03 mg/L), FeSO_4_·7H_2_O (0.20 mg/L), CuSO_4_·5H_2_O (0.03 mg/L), CoCl_2_·6H_2_O (0.10 mg/L), MnSO_4_·H_2_O (0.10 mg/L), Na_2_MoO_4_·2H_2_O (0.04 mg/L), H_3_BO_3_ (0.03 mg/L)				

Note*: suitable for pilot-scale experiment, ** suitable for batch test and pilot-scale experiment, SDP: spatial denitrification performance test, LDL: limit denitrifying load test.

The test methods employed were as follows: nitrate, nitrite, and ammonia levels were determined using a UV spectrophotometer (Hach, DR-5000) in accordance with Chinese Environmental Standards (HJ/T 346-2007, GB 7493-87, HJ 535-2009), respectively, COD was measured using the acidic potassium dichromate oxidation method (DRB-200, Hach Corporation, United States), SS was determined via the gravimetric method (GB 11901-89), pH values were monitored using a pH-201 m (Hanna Corporation, Italy), dissolved oxygen (DO) and temperature were monitored with a portable DO meter (JPB-607A). Degradation curves were fitted with linear kinetics and pseudo-1^st^-order kinetics to describe the mechanism of nitrogen removal (Deng and Wheatley, 2018). The fitting function of pseudo-1^st^-order model used $y=y_{0}+A_{1}e^{-\frac{x}{t_{1}}}$ in origin 2020b.

### 2.2 Nitrate nitrogen degradation curve for mature biofilm

**FIGURE 1:**
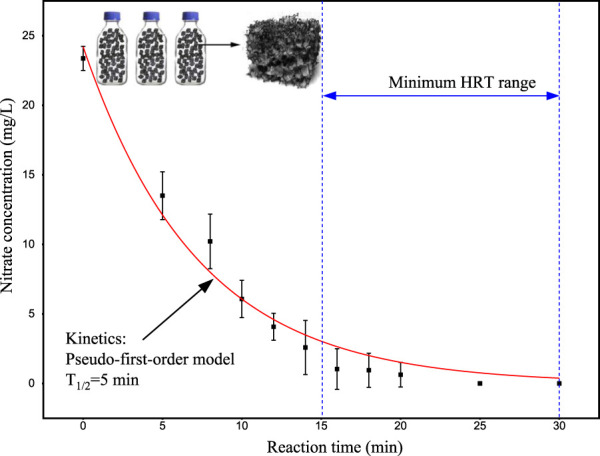
Nitrate removal kinetic of mature biofilm Experiment condition: 25°C, pH 7.5, initial NO_3_^−^-N = 25 mg/L, C/N/P = 100:20:1.

Before the experiment, batch tests were conducted to investigate the dynamics of NO_3_^−^-N degradation using mature biofilm. The mature biofilm (see Supplementary Figure A1) was obtained from an I-BAF reactor that had been operational for 6 months. This biofilm exhibited efficient denitrification capability, which could determine the minimum HRT of the I-BAF system. The higher the denitrifying capability, the lower the HRT could be set. The mature biofilm, covered with denitrifying bacteria, was placed into three conical bottles (500 mL each) with a filling ratio of 95%. The composition used in this section is outlined in Table 1. The nitrate degradation curve was measured over time, as shown in Figure 1, indicating that nitrate degradation followed pseudo-1^st^-order kinetics with a half-life (T_1/2_) of 5 min. Nitrate nitrogen at a concentration of 25 mg/L was degraded to near 0 mg/L within 15–30 min, suggesting that the minimum HRT could be within this range when operating the continuous-flow I-BAF system.

### 2.3 Lab-scale and pilot-scale I-BAF setup

As depicted in Supplementary Figure A2, lab-scale continuous-flow experiments were conducted in an organic glass cylindrical reactor (BAF_L_) with dimensions of 15 cm (inner diameter) by 75 cm (height), having a working volume of 13.2 L. The reactor was packed with polyurethane foam (PUF) carriers in the form of 3 cm × 3 cm × 3 cm cubes, possessing a specific surface area ranging from 80 to 120 m^2^/g and a wet density of 1 g/cm^3^, packed to a 95% filling ratio (Ye et al., 2005). Synthetic wastewater was continuously pumped from the bottom through the bioreactor using a peristaltic pump (BT100s, China). Nitrogen (N_2_) was introduced into the bottom of the reactor to regulate dissolved oxygen (DO) concentration and promote uniform water quality within the biofilm system. Four water sampling ports were positioned at 150 mm intervals along the height of the reactor, with the effluent port located at a height of 750 mm. Additionally, four biofilm-carrier sampling ports were situated on another side for microbial diversity analysis (Supplementary Figure A2).

As shown in Figures 6C, D, pilot-scale continuous-flow experiments were carried out in an organic glass cubic reactor (dimensions = 1 m × 1 m × 1 m) with the working volume of 500 L. PUFs carriers and commercial hollow ball (CHB) carriers packed the two reactors respectively at 95% filling ratio. The synthetic wastewater was continuously driven from the bottom into the bioreactors. Air was pumped into the bottom of the reactors to regulate DO concentration. Four water sampling ports (S1–S4) were set respectively every 120 mm height, and the effluent was discharged by tumbling bay. The bottom of the reactor was equipped with backwash system.

### 2.4 Experimental process and operating conditions

The composition and corresponding concentrations of the lab-scale experiment are listed in Table 1. A denitrifying microbial consortium (DMC) weighing 13.2 g was added to the BAF_L_ and operated aerated with N_2_ for 48 h to allow the denitrifying bacteria to adapt to the environment (adaptation period). Following the 48-h adaptation period, the continuous-flow experiment proceeded to assess the denitrifying performance of the BAF_L_. The HRT was regulated to 6, 4, 2, 1, 0.5, and 0.25 h, as depicted in Supplementary Figure A3. Water samples were collected daily from the inlet and outlet to measure four indices (COD, nitrate, nitrite, ammonia). Furthermore, spatial denitrification performance (SDP) tests were conducted under HRTs of 6, 4, 2, 1, 0.5, and 0.25 h (Supplementary Figure A3). Subsequently, the limit denitrifying load (LDL) test was explored by increasing the influent nitrate concentration to 50 mg/L and 100 mg/L (Supplementary Figure A3). Water samples were collected daily from the inlet and outlet to measure COD, nitrate, nitrite, and ammonia concentrations.

For the pilot-scale experiment, 800 g of the inoculum strain was added into BAF_1_ and BAF_2_, respectively. The composition and corresponding concentrations are detailed in Table 1. The adaptation period and continuous-flow experiment were conducted as shown in Supplementary Figure A3, with air aeration. Water samples were collected daily from both the inlet and outlet. Additionally, four samples were taken at water sampling ports (S1–S4) every 2 days when the HRT was set to 1 h.

### 2.5 Microbial community analysis

Four biofilm samples were collected from biofilm carrier sampling ports (see Supplementary Figure A2) in BAF_L_ during the stable operation phase (30th day, HRT = 0.5 h). Additionally, three biofilm samples were randomly collected from both BAF_1_ and BAF_2_ during the stable operation phase (64th day, HRT = 0.5 h). These samples, along with the inoculation strain, were collected and stored at 4°C for microbial community analysis (Liu et al., 2021).

## 3 Results and discussion

### 3.1 The start-up of biofilter

**FIGURE 2:**
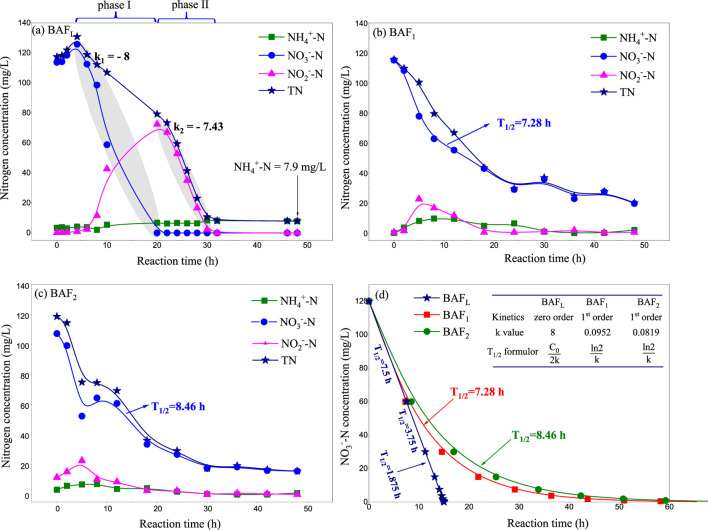
Composition change curve in adaptation stage of immobilized microbial filter **(A)** Lab-scale I-BAF, **(B)** Pilot-scale I-BAF loaded with PUFs carriers, **(C)** Pilot-scale I-BAF loaded with CHB carriers, **(D)** Simulated kinetic curve of nitrate degradation.

The spontaneous formation of the active layer in biological filters typically takes anywhere from a week to several months (Jing et al., 2012; Štembal et al., 2004; Bosander and Westlund, 2000). Generally, the duration of biofilter start-up is primarily determined by the inoculated microorganisms (Breda et al., 2019), the type of carrier (Smet et al., 1996), and the aeration patterns (Bacquet et al., 1991). Aeration patterns can be categorized into three forms: anaerobic, aerobic, and intermittent aeration. The aeration mode utilized in our lab-scale biofilter was an anaerobic pattern using nitrogen gas. As depicted in Figure 2A, nitrate decreased to zero within the initial 20 h (phase I), while nitrite reached its maximum concentration (72.4 mg/L) at this point. Such a phenomenon is commonly observed during the initial operation process of biological denitrifying filters (Gan et al., 2020). Initially, most of the nitrate is transformed into nitrite, which then gradually degrades into N_2_O and eventually into nitrogen gas (Das et al., 2021). In Figure 2A, the NO_3_^−^-N degradation rate constant (k_1_ = - 8) can be considered as the NO_2_^−^-N generation rate constant during phase I. This constant was faster than the nitrite degradation constant (k_2_ = −7.43), resulting in its accumulation during phase I (Zhang et al., 2020; Yue et al., 2009).

It is noteworthy that both the degradation of nitrate and nitrite conformed to linear dynamics (0-order kinetics) during the adaptation phase, a finding which is also consistent with Yao’s research (Yao et al., 2022). They suggested that TN removal follows zero-order kinetics in the presence of NH_4_^+^ during the initial nitrogen removal stage. Occasionally, ammonia accumulated gradually to 7.9 mg/L throughout the entire adaptation phase, as shown in Figure 2A.

The aeration mode of the pilot-scale biofilter was an aerobic pattern using air (Zupanèiè et al., 2008). As depicted in Figures 2B, C, nitrate degradation followed a pseudo-1^st^-order kinetics model, with half-life periods (T_1/2_) of BAF_1_ and BAF_2_ being 7.28 h and 8.46 h, respectively. This indicates that their denitrifying rates were significantly lower than that of BAF_L_. This suggests that the anaerobic aeration pattern is more conducive to the growth of denitrifying bacteria (Hong et al., 2019).

In Figure 2D, the half-life period (T_1/2_) of BAF_L_ exhibited a linear relationship with the initial nitrate nitrogen concentration. At a concentration of 25 mg/L, the T_1/2_ of BAF_L_ was 1.56 h, significantly larger than that of the mature biofilm (T_1/2_ = 5 min, as mentioned in Section 2.2). This indicates that the denitrifying capacity of the mature biofilm was 18.7 times higher than that of the initial biofilm.

### 3.2 Denitrification performance of lab-sacle I-BAF

**FIGURE 3:**
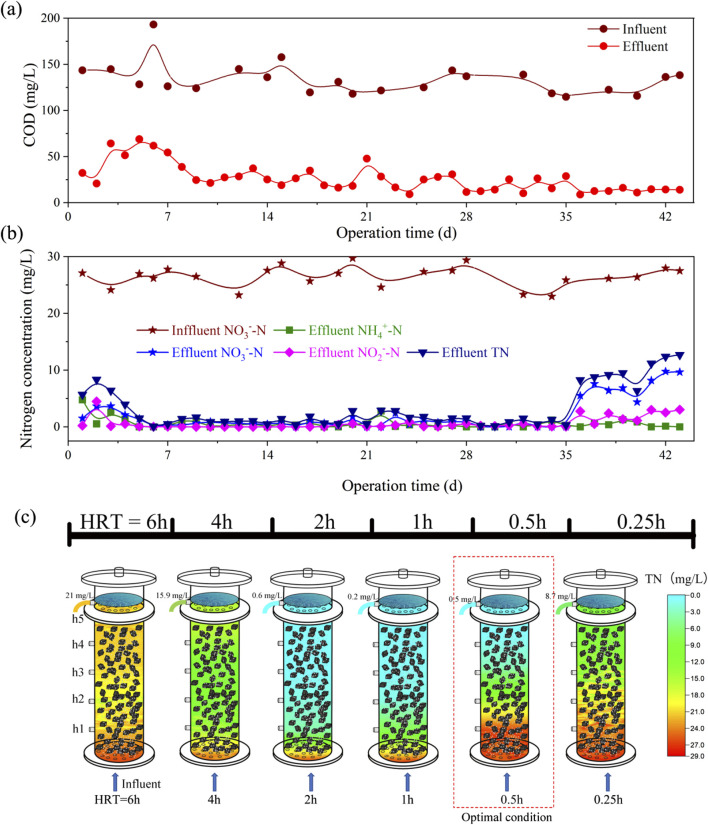
Nitrogen removal performance and TN spatial distribution of lab-scale I-BAF **(A)** COD removal performance, **(B)** Nitrogen removal performance, **(C)** Spatial distribution of TN with different HRT.

The denitrification performance of a biofilter is mainly reflected in the effluent quality, organic load, and denitrifying load (Wu et al., 2021). In Figures 3A, B, concerning COD and TN, the continuous-flow test indicated that the lab-scale biofilter was not initially stable (at HRT = 6 h), suggesting that the denitrifying bacteria were still in the start-up stage (Jing et al., 2012). During this phase, both TN and COD levels in the effluent remained high, with COD exceeding the Chinese Discharge Standard of Pollutants for Municipal Wastewater Treatment Plants (GB 18918-2002, Level A < 50 mg/L).

Figure 3B shows that TN levels ranged from 0.2 to 2.8 mg/L between the 7th and 35th day (corresponding to an HRT of 4 to 0.5 h), with an average of 1.2 mg/L. Concurrently, the effluent COD remained below 50 mg/L (as shown in Figure 3A). The water quality, with TN at 1.2 mg/L, was near the limit of biological nitrogen removal (Oleszkiewicz and Barnard, 2006). In China, TN values below 1 mg/L are even regulated as drinking water source standards (GB3838-2002). The achievement of ultra-low TN emission was attributed to the advantages of heterotrophic denitrification by immobilized microorganisms, wherein various dominant microbial populations could acclimate and rapidly propagate (Gan et al., 2020).

Continuing the operation of the I-BAF for over a month resulted in the complete formation of mature biofilm, as depicted in Supplementary Figure A1, which appeared in a colloidal state with a milky white appearance. The time from microbial inoculation to biofilm maturation typically takes about 2–3 weeks. The duration of biofilm maturation varies depending on factors such as inoculum type, media, nutrients, and temperature. Visually, mature biofilms display a significant amount of gel-like active microorganisms firmly attached to the carrier. Moreover, reactors with mature biofilms can quickly remove nitrogen compounds (25 mg/L to 1 mg/L) in a very short time (0.5 h).

Figure 3C illustrates the spatial distribution of TN in the mature biofilm I-BAF. In this depiction, cooler colors represent lower TN concentrations, while warmer colors indicate higher concentrations. The effluent TN concentrations were recorded as 21, 15.9, 0.6, 0.2, 0.5, and 8.7 mg/L at HRT of 6, 4, 2, 1, 0.5, and 0.25 h, respectively. Notably, the effluent quality achieved at HRT of 2 to 0.5 h met the target of TN < 1 mg/L. Particularly, an HRT of 0.5 h was deemed optimal as it achieved an effluent TN of 0.5 mg/L while maximizing the utilization of the biofilter. To the best of our knowledge, the minimum HRT achieved in biofilters for denitrification is 1.5 h (Cao et al., 2014). Thus, further reductions in HRT could lead to savings in building costs and area requirements. Conversely, some studies have set HRT to more than 6 h (Chen et al., 2019; Ryu and Lee, 2009), a practice deemed improper during the stable operation stage of mature biofilm based on our research findings.

**FIGURE 4:**
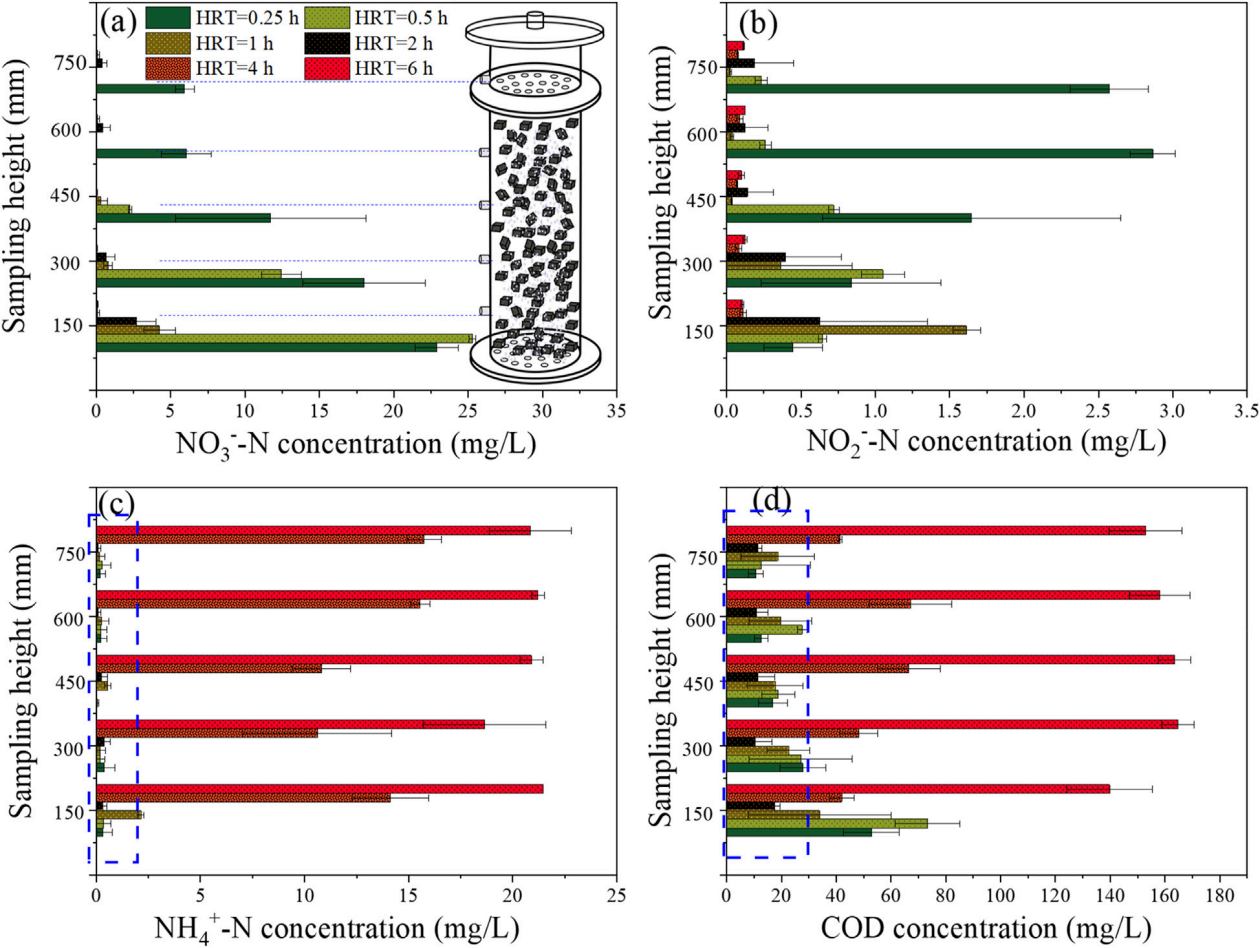
Spatial distribution of **(A)** nitrate, **(B)** nitrite, **(C)** ammonia and **(D)** COD under different HRT.

The batch test conducted in Section 2.2 suggested that the minimum HRT might fall within the range of 0.5−0.25 h. However, the continuous-flow operation of the I-BAF (Figure 3) revealed that achieving an HRT of 0.25 h was challenging (with an average TN concentration of 9.8 mg/L) due to the hydraulic load exceeding the maximum capacity of BAF_L_. It is worth noting that a larger HRT was also not suitable for nitrogen removal (e.g., effluent TN concentration of 21 mg/L at HRT 6) due to the accumulation of ammonia at this point. Figure 4 illustrated the spatial distribution of three types of nitrogen. Ammonia remained at high concentrations (ranging from 10.6 to 21.5 mg/L) when HRT was between 4 and 6 h (Figure 4C), indicating that most nitrate was converted to ammonia nitrogen instead of nitrogen gas. Additionally, Figure 4D demonstrated that larger HRTs (4–6 h) also resulted in COD levels exceeding standards (GB 18918-2002).

Figure 4A demonstrates that NO_3_^−^-N could be effectively removed under HRTs ranging from 6 to 0.5 h, with the exception of 0.25 h. The spatial distribution of nitrite is illustrated in Figure 4B. Notably, when the HRT was 0.25 h, the effluent nitrite concentration remained at 2.6 mg/L (at a height of 750 mm). Such level of nitrite concentration is still highly toxic to aquatic organisms (Lewis and Morris, 1986). Nitrite was also observed to be generated at other HRTs, such as 0.5–6 h, but it could be degraded to lower concentrations in the effluent. The results indicate that the biofilter undergoes a process of nitrite accumulation followed by degradation as the sampling height increases (Qiu and Ma, 2005).

### 3.3 Maximum denitrifying load in lab-scale I-BAF

**FIGURE 5:**
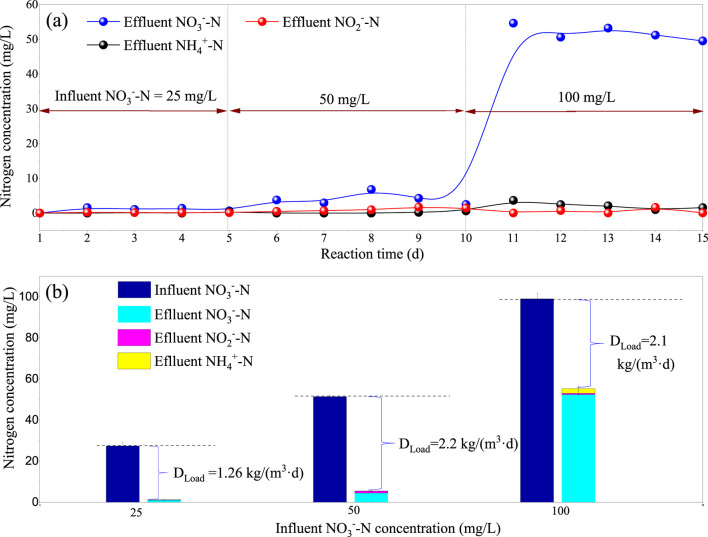
Effluent index **(A)** and denitrification load **(B)** D_load_ under influent nitrate nitrogen concentrations of 25, 50, and 100 mg/L.

The previous tests indicated that the optimal condition was an HRT of 0.5 h. This section aimed to explore the maximum denitrifying load using higher NO_3_^−^-N concentrations in the influent (50 mg/L and 100 mg/L). As shown in Figure 5A, increasing the influent NO_3_^−^-N concentration to 100 mg/L resulted in the deterioration of effluent quality, with NO_3_^−^-N reaching 54.6 mg/L. Figure 5B calculated the denitrifying load (D_load_) at three stages, resulting in values of 1.26 kg (TN)/(m^3^·d), 2.2 kg (TN)/(m^3^·d), and 2.1 kg (TN)/(m^3^·d) respectively. The D_load_ peaked at 2.2 kg (TN)/(m^3^·d) when the influent nitrate concentration was 50 mg/L, with the effluent quality slightly higher (NO_3_^−^-N = 4.4 mg/L). However, the load remained unchanged as the influent nitrate concentration increased from 50 mg/L to 100 mg/L. Therefore, the I-BAF described in this article is suitable for influent nitrate concentrations ranging from 0 to 50 mg/L in wastewater treatment applications (HRT = 0.5 h).

When influent nitrate nitrogen increased from 50 to 100 mg/L, the filter was punctured, and D_load_ did not continue to rise, indicating that the maximum nitrogen removal load of the filter was 2.2 kg/(m^3^·d). Other conditions: HRT = 0.5 h, T = 25°C, pH = 7.5.

Table 2 presents the denitrifying load and water quality parameters from relevant studies. Wang et al. achieved a high denitrifying load of 0.84 kg (TN)/(m^3^·d) when treating effluent from the secondary sedimentation tank, although the effluent TN concentration was slightly higher at 10.3 mg/L (Wang et al., 2014). In contrast, Han’s research achieved a lower effluent TN concentration (5.75 mg/L), but their denitrifying load was just 0.04 kg (TN)/(m^3^·d) (Han et al., 2015). Few studies have examined the relationship between the lowest effluent TN and the maximum denitrifying load in actual biofilters. Table 2 demonstrates that both the denitrifying load and effluent quality in our research were superior to current technologies. In this study, we explored the limit parameters. By setting the HRT to 0.5 h and influent TN to 50 mg/L, we achieved the maximum denitrifying load of 2.2 kg (TN)/(m^3^·d). Furthermore, under an HRT of 0.5 h and influent TN of 25 mg/L, the I-BAF could treat up to 48 times its own volume of wastewater every day while maintaining an effluent TN concentration below 1 mg/L.

**TABLE 2:** Comparison of main parameters of biofilter.

Reference	Carbon source	COD/N	Influent TN (mg/L)	Effluent TN (mg/L)	Media	Actual HRT (h)	Denitrifying load kg (TN)/(m^3^·d)
Wang et al. (2014)	Liquor wastewater	3.5	68.9–80.7	10.3	sand	2	0.84
Han et al. (2015)	Ethanol	—	30	5.75	bagasse	14	0.04
Li et al. (2010)	—	11.6	26.8	12.1	volcanic rock	6	0.06
Cao et al. (2014)	methanol	5	30.8	10	Quartz sands	1.5	0.33
Gómez et al. (2002)	—	6–10	39–56	<15	Ceramsite	9.12	0.11
This paper	Glucose	5	25	<1	PUFs	0.5	1.26
This paper	Glucose	5	25	<8.7	PUFs	0.25	1.56
This paper	Glucose	5	50	<4	PUFs	0.5	2.2

Note: Based on the literature review, other studies reported influent TN, concentrations ranging from 26.8 to 80.7 mg/L, with effluent TN, levels between 5.75 and 12.1 mg/L. The maximum nitrogen removal load (D_load_) achieved was 0.84 kg (TN)/(m³·d). In this study, the influent TN, is approximately 25 mg/L, with effluent TN, reaching 4 mg/L, resulting in a D_load_ of 2.2 kg (TN)/(m³·d), which is currently the highest known value.

### 3.4 Denitrification performance and load analysis of pilot-sacle I-BAF

**FIGURE 6:**
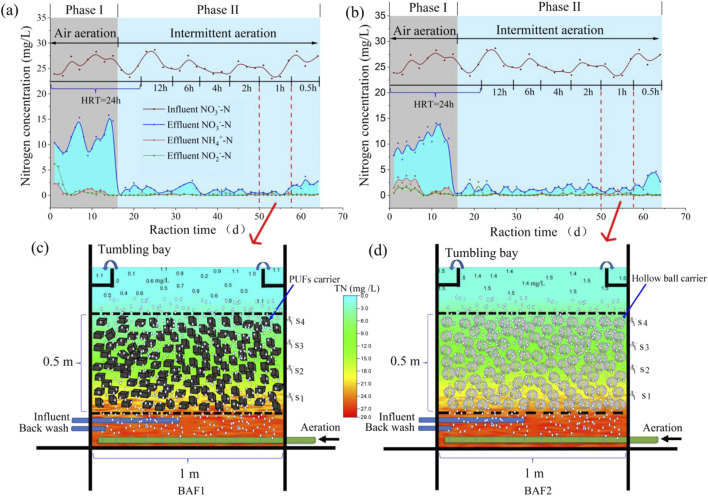
**(A)** Nitrogen removal performance of I-BAF packed with PUFs **(A)** and CHB **(B)**. TN spatial distribution of PUFs I-BAF **(C)** and CHB I-BAF **(D)** when HRT was 1 h.

Figure 6 illustrates the nitrogen removal performance of the pilot-scale I-BAF (active volume = 0.5 m^3^). The continuous-flow experiment was divided into two phases: phase I (15 days) and phase II (49 days). Figures 6A, B indicate that nitrate levels during phase I remained high (10–15 mg/L), ultimately resulting in elevated TN concentrations. The primary reason for the low denitrifying efficiency was the fluctuation in dissolved oxygen (DO) levels in BAF_1_ and BAF_2_, ranging between 2.2 and 7.5 mg/L, which was not conducive to the survival of denitrifying bacteria (Gómez et al., 2002). These results suggest that ordinary air aeration alone could not achieve ultra-low nitrogen emissions (1 mg/L). To address this issue, intermittent aeration (30 min on, 30 min off) was adopted in phase II (Zhang et al., 2017). The results of phase II demonstrated that effluent nitrate quickly dropped to 1 mg/L, achieving the same water quality as observed in the lab-scale test (using N_2_ aeration). The 30-min aeration and 30-min no aeration pattern were determined based on the nitrate removal rate (Figure 1), which indicated that the mature biofilm could effectively remove nitrate within 30 min. Subsequent tests showed that TN levels remained relatively stable under different HRTs (12, 6, 4, 2, 1 h). However, at an HRT of 0.5 h, the nitrate concentration in the pilot-scale system increased slightly to a higher level (2.5 mg/L) compared to the lab-scale test (1.2 mg/L).

Figures 6C, D illustrates the spatial distribution profile of TN at an HRT of 1 h. It demonstrates that both BAF_1_ and BAF_2_ achieved ultra-low nitrate emissions. The effluent quality of BAF_1_ (1.1 mg/L) was superior to that of BAF_2_ (1.5 mg/L), indicating that the PUFs carrier performed better than the CHB carrier. Additionally, the SS in BAF_1_ (3.6 mg/L) was significantly lower than that in BAF_2_ (32.4 mg/L), with the latter exceeding Chinese standards (10 mg/L). Generally speaking, the I-BAF with PUFs was more conducive to biofilm culturing, resulting in clearer effluent water compared to the I-BAF with CHBs.

In summary, this study successfully achieved high-efficiency denitrification (TN near 1 mg/L) and obtained the maximum denitrifying load (denitrifying load = 1.26–2.2 kg (TN)/(m^3^·d)) in lab-scale experiments. Pilot-scale tests also achieved effluent TN concentrations of 1.1–1.5 mg/L at the minimum HRT of 1 h. The results from both lab-scale and pilot-scale tests could significantly reduce the facility construction costs of advanced wastewater treatment.

### 3.5 Microbial community analysis

**FIGURE 7:**
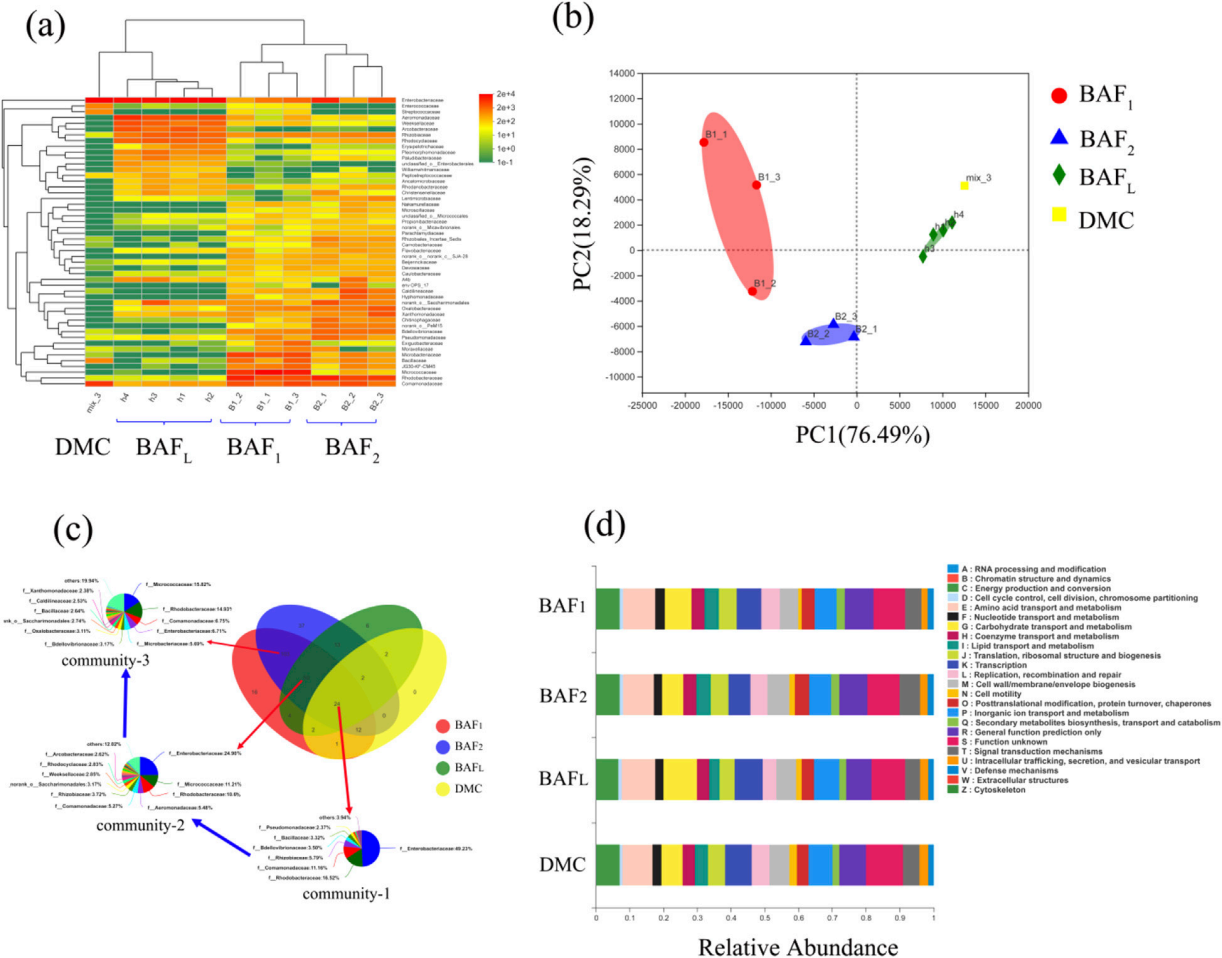
Microbial diversity analysis of community, **(A)** Community heatmap analysis on family level, **(B)** Principal components analysis on family level, **(C)** Species succession analysis, **(D)** COG function classification.

Microbial community analysis is essential for understanding the composition and dynamics of microbial populations, which directly influence the efficiency of nitrogen removal processes. As highlighted by Du et al. (2016), such analyses offer valuable insights into the underlying principles governing nitrogen removal in biological systems. In this study, principal components analysis (PCA) was employed to categorize the samples into four distinct groups, as shown in Figure 7B. This classification reveals that samples originating from the same biological filter exhibit similar microbial community structures, indicating a degree of uniformity in microbial interactions and functional capabilities within individual systems. Furthermore, it is important to note that these microbial structures can evolve over time, particularly during the cultivation process in the integrated biological aerated filter (I-BAF). Variations in microbial composition may arise due to changes in operational conditions, nutrient availability, or external environmental factors.

In Figure 7A, the analysis of the denitrifying microbial community (DMC) reveals that it primarily consists of three genera: *Enterobacter*, *Comamonas*, and *Bacillus*. This relatively simple composition indicates that the DMC used for inoculation contains relatively few types of denitrifying functional bacteria.

On the other hand, the biological aerated filter with high hydraulic loading (BAF_L_) displays a more complex microbial diversity, predominantly containing *Enterobacter*, *Aeromonas*, *Comamonas*, and *Rhodococcus* (The microbial species with the abundance greater than e^2 exceed 20 types). This diversity suggests that BAF_L_ offers a more favorable environment for various microbial interactions, which can enhance the overall efficiency of nitrogen removal processes. Notably, BAF_1_ and BAF_2_ exhibited greater microbial similarity, primarily featuring *Rhodococcus*, *Comamonas*, *Micrococcus*, and *Enterobacter*, indicating that these systems may operate under similar conditions and support similar microbial populations.

The principal components analysis of BAF_L_ reveals minimal differences in microbial diversity within the spatial distribution of the mature biofilm (Figure 7B), suggesting a stable and well-established community structure. *Enterobacter* consistently appeared across all four groups, a finding that aligns with previous studies indicating its role as a highly tolerant denitrifying bacterium (Ma et al., 2021). Furthermore, a comparison of microbial diversity shows that the mature biofilm, including BAF_L_, BAF_1_, and BAF_2_, contains a broader variety of microbial species than the DMC, as illustrated in Supplementary Figure A4. This observation highlights the significant proliferation of microorganisms during the biofilm culture process, which is crucial for optimizing bioreactor performance. The increased microbial diversity within the biofilm is likely to enhance the functional capabilities of the system, thereby improving nitrogen removal efficiency and overall treatment outcomes.

Figure 7C illustrates the presence of *Enterobacter*, *Micrococcus*, and *Rhodococcus* across all four groups, highlighting the capability of these three genera to perform both aerobic and anaerobic denitrification, as supported by various studies (Chen et al., 2012; Mpongwana et al., 2000; Tait, 1975). This versatility is crucial for optimizing nitrogen removal processes in varying environmental conditions. Notably, *Rhodococcus* was more identified in the biofilters (BAF_L_, BAF_1_, BAF_2_) instead of DMC, suggesting that this genus may have been introduced and established during the culture process, as indicated by Sun et al. (2021). This observation underscores the adaptive nature of microbial communities in response to specific operational conditions.

Despite the differences in microbial composition, the four groups displayed similar functional metabolic pathways based on COG pathway analysis. The primary functions identified were related to essential microbial processes, including carbohydrate transport and metabolism, energy production, and transcription, as depicted in Figure 7D. This functional consistency across diverse microbial communities indicates that, while the composition may vary, the fundamental metabolic capabilities necessary for effective denitrification remain intact. In conclusion, *Enterobacter* emerges as the dominant denitrifier in both anaerobic and aerobic processes, followed closely by *Comamonas* and *Rhodococcus*. This dominance suggests that enhancing the growth of these key microbial populations could significantly improve nitrogen removal efficiency in wastewater treatment systems. Additionally, the abundance of the aforementioned three types of denitrifying bacteria could serve as a criterion for assessing the denitrification capacity of future biological filters.

## 4 Conclusion

The lab-scale and pilot-scale I-BAF were constructed to investigate denitrification performance and maximum denitrifying load. During the adaptation phase, nitrite would accumulate within the initial 20 h. In the lab-scale I-BAF, the nitrate degradation process during the adaptation stage followed zero-order kinetics, while during the continuous-flow stage, it conformed to pseudo-first-order kinetics. The maximum denitrifying load was 2.2 kg (TN)/(m^3^·d) under conditions of influent NO_3_^−^-N = 50 mg/L and HRT = 0.5 h, achieving an effluent TN of 4.4 mg/L. The optimal combination was influent NO_3_^−^-N = 25 mg/L and HRT = 0.5 h, which could achieve a water quality of TN < 1 mg/L. Spatial distribution tests of mature biofilm revealed that the proper HRT ranged from 2 h to 0.5 h. Longer HRT led to ammonia accumulation, while much shorter HRT caused effluent NO_2_^−^-N levels to exceed 2.6 mg/L. Microbial diversity analysis showed that the genera *Enterobacter* were dominant in all denitrifying systems, followed by *Comamonas* and *Rhodococcus*, and the abundance of the aforementioned three types of denitrifying bacteria could serve as a criterion for assessing the denitrification capacity of future biological filters. This study combined batch tests and continuous-flow experiments to explore ultra-low TN emissions, providing support for further improvements in advanced wastewater treatment in future plants.

## Data Availability

The raw data supporting the conclusions of this article will be made available by the authors, without undue reservation.
