# Supplementary material for: Optimizing nitrogen removal in advanced wastewater treatment using biological aerated filters
Source: Front Bioeng Biotechnol. 2024 Nov 28;12:1463544. doi: 10.3389/fbioe.2024.1463544 (PMC11635964; doi:10.3389/fbioe.2024.1463544)
Supplement: Supplementary file 1 [file DataSheet1.docx]

Supplementary Materials


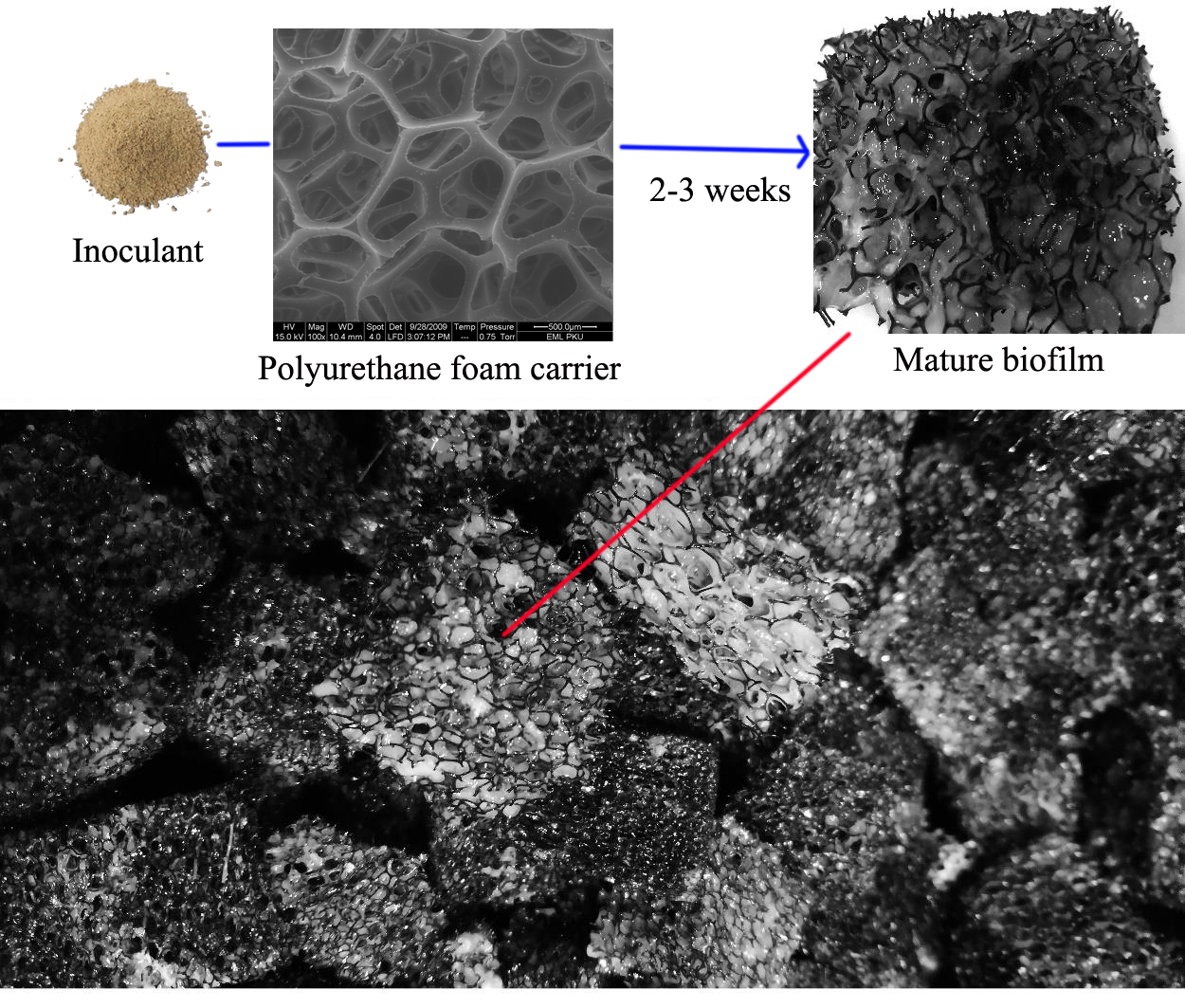


**Figure A1.** Graph of mature biofilm (BAF_L_)


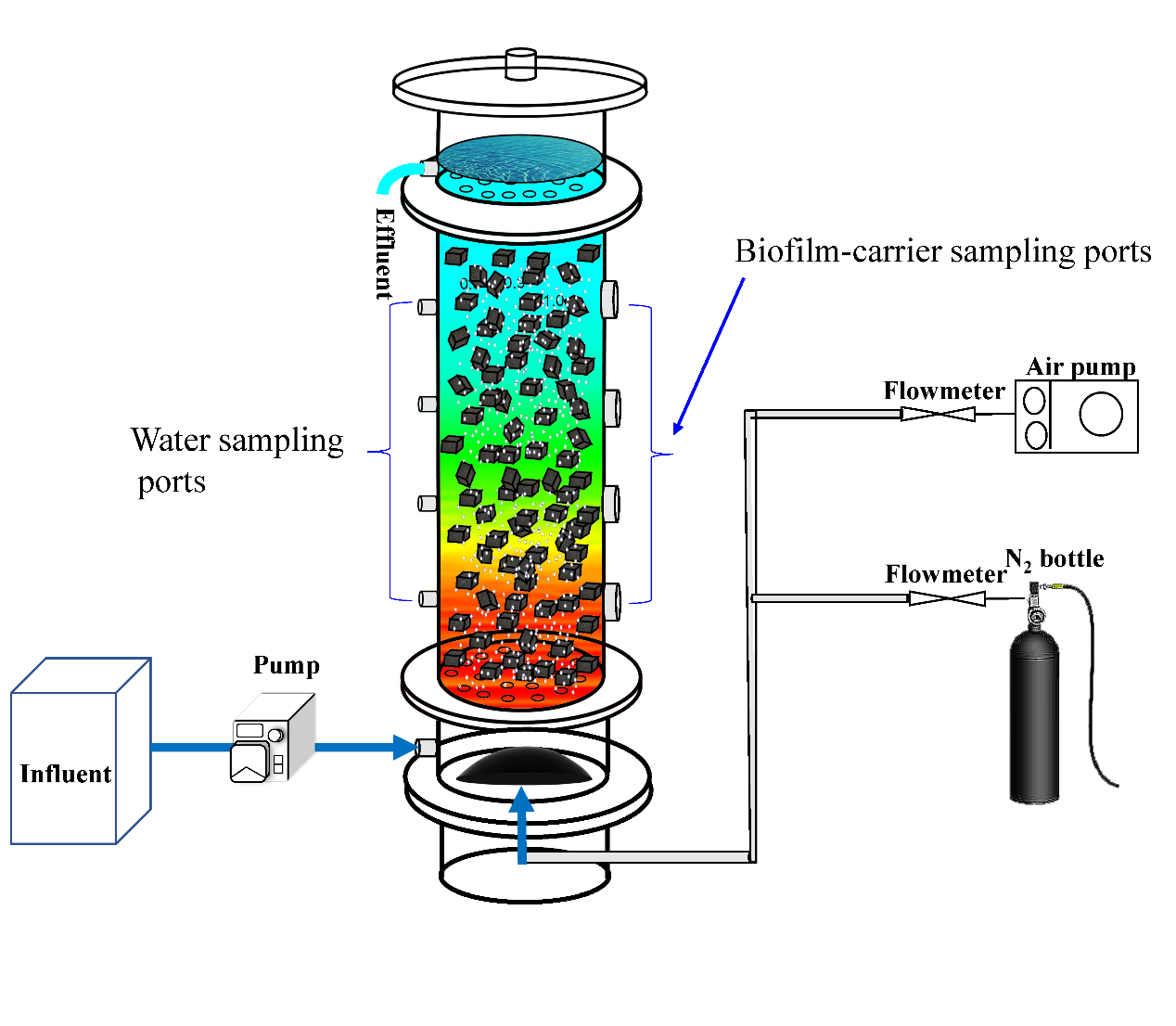


**Figure A2.** Lab-scale I-BAF reactor


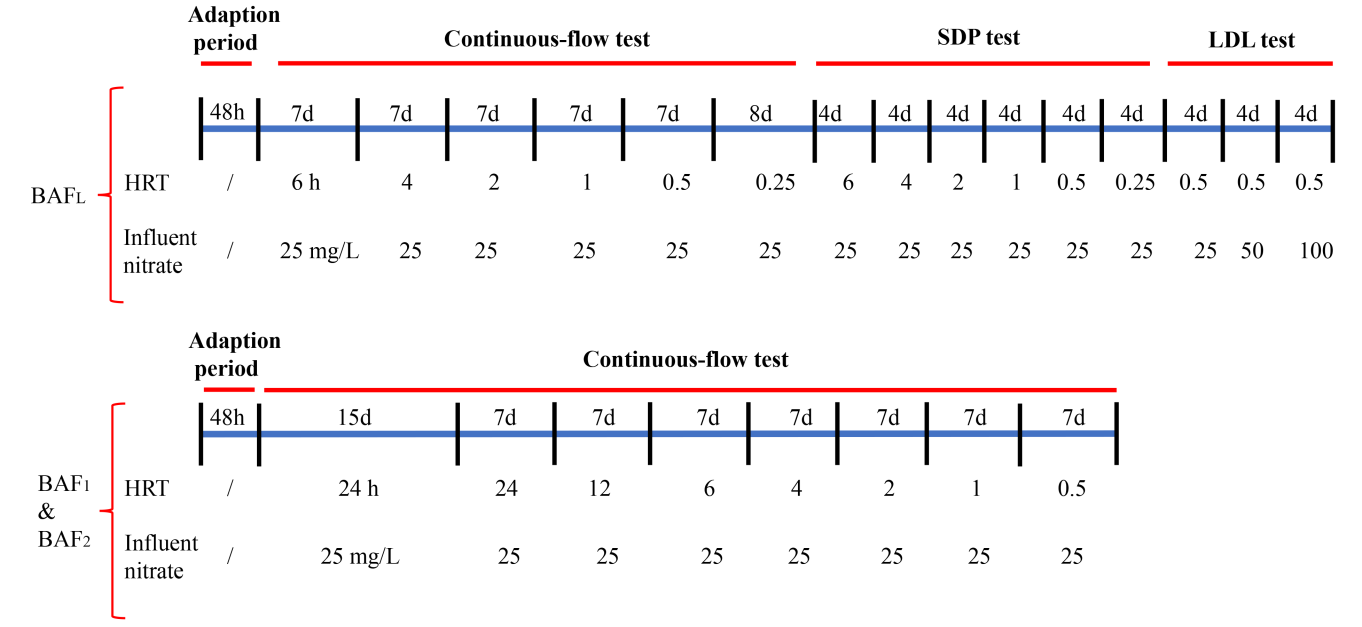


**Figure A3.** [Schematic](javascript:;) [diagram](javascript:;) of experimental process


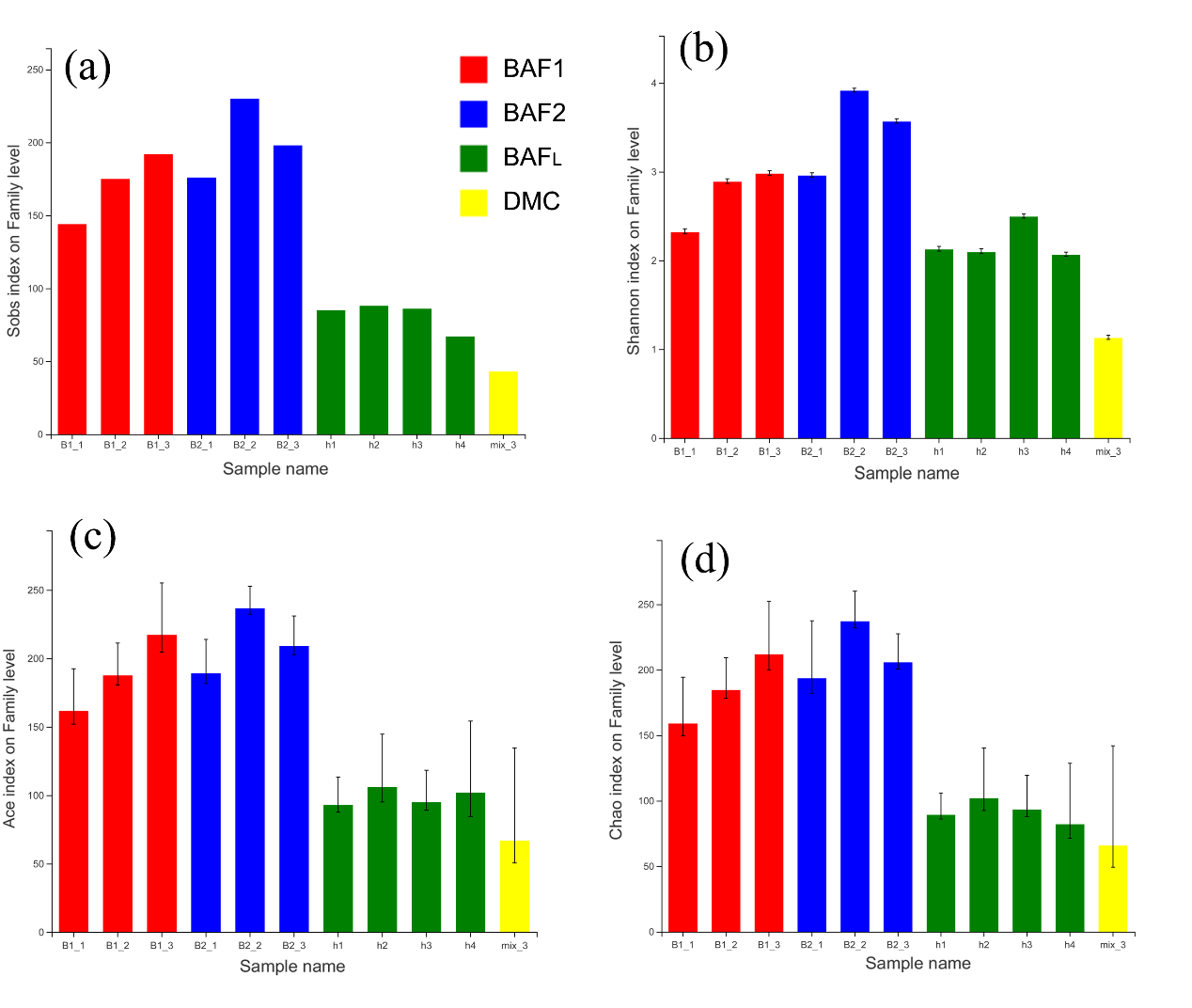


**Figure A4.** Alpha diversity estimator
